# Integrated matrix-assisted laser desorption ionization time-of-flight mass spectrometry serotype-specific peak analysis and *trans*-cinnamic acid antimicrobial activity against *Salmonella* spp. from the poultry chain

**DOI:** 10.14202/vetworld.2026.642-653

**Published:** 2026-02-23

**Authors:** Nuttanit Jirapanth, Patamaporn Umnahanant, Kitiya Vongkamjan, Juree Tuangrithaiwanich, Sirijanya Rakmit, Nawin Thongdee, Arsooth Sanguankiat

**Affiliations:** 1 Graduate Program in Animal Health and Biomedical Sciences, Faculty of Veterinary Medicine, Kasetsart University, Bangkok 10900, Thailand; 2 Department of Veterinary Technology, Faculty of Veterinary Technology, Kasetsart University, Bangkok 10900, Thailand; 3 Department of Biotechnology, Faculty of Agro-Industry, Kasetsart University, Bangkok 10900, Thailand; 4 Kamphaeng Saen Veterinary Diagnostic Center, Faculty of Veterinary Medicine, Kasetsart University, Kamphaeng Saen, Nakhon Pathom 73140, Thailand; 5 Department of Veterinary Public Health, Faculty of Veterinary Medicine, Kasetsart University, Kamphaeng Saen, Nakhon Pathom 73140, Thailand

**Keywords:** antimicrobial resistance, food safety, MALDI-TOF MS, poultry chain, *Salmonella*, serotype identification, specific peak analysis, *trans*-cinnamic acid

## Abstract

**Background and Ai:**

*Salmonella* spp. are major foodborne pathogens within the poultry chain and pose a substantial public health risk. Rapid and accurate serotype identification is essential for effective surveillance, outbreak investigation, and control strategies. Matrix-assisted laser desorption ionization time-of-flight mass spectrometry (MALDI-TOF MS) provides rapid species-level identification; however, its reliability for serotype differentiation remains limited by overlapping spectral profiles and incomplete reference databases. Concurrently, rising antimicrobial resistance (AMR) necessitates exploration of alternative antimicrobial agents. This study aimed to (i) evaluate the accuracy of MALDI-TOF MS for *Salmonella* serotype identification using specific peak analysis compared with conventional serotyping and (ii) assess the antimicrobial effectiveness of *trans*-cinnamic acid (TCA) against poultry-associated *Salmonella* isolates.

**Materials and Method:**

A total of 63 *Salmonella* isolates representing six serotypes were analyzed, including *Salmonella* Enteritidis, *Salmonella* Typhimurium, *Salmonella* Kentucky, *Salmonella* Newjersey, *Salmonella* Fresno, and *Salmonella* Weltevreden, obtained from poultry production environments in Thailand. MALDI-TOF MS performance was evaluated against conventional serotyping using overall percentage agreement (OPA), positive percentage agreement (PPA), negative percentage agreement (NPA), and Cohen’s kappa statistic. Serotype-specific mass spectral peaks were identified through comparative analysis with reference strains. The antimicrobial activity of TCA was evaluated using broth dilution assays to determine the minimum inhibitory concentration (MIC) and minimum bactericidal concentration (MBC).

**Result:**

MALDI-TOF MS showed high concordance with conventional serotyping, with OPA, PPA, and NPA values ranging from 97.3% to 100%. Cohen’s kappa values indicated substantial to perfect agreement, with minor discordance observed for *S*. Enteritidis (κ = 0.65). Serotype-associated peaks were consistently detected at 6,094 ± 2 mass-to-charge ratio (m/z) for *S*. Enteritidis, 7,156 ± 2 m/z for *S*. Typhimurium, and 5,370 ± 2 m/z for *S*. Kentucky. TCA exhibited uniform antimicrobial activity against all tested serotypes, with MIC and MBC values of 10 mM and 20 mM, respectively, and no significant differences among serotypes (p > 0.05).

**Conclusio:**

MALDI-TOF MS combined with specific peak analysis provides a reliable and rapid approach for *Salmonella* serotype identification in the poultry chain, although database expansion remains necessary for uncommon serotypes. TCA demonstrated consistent inhibitory and bactericidal activity, supporting its potential role as a complementary, non-antibiotic intervention for *Salmonella* control and AMR mitigation in poultry production systems.

## INTRODUCTION

*Salmonella* is a major foodborne pathogen and a significant public health concern. The primary route of *Salmonella* transmission is through the consumption of contaminated food or water [1–3]. Salmonellosis is among the top 10 causes of gastrointestinal diseases in Southeast Asia, with approximately 16 million cases and 16,000 deaths annually [4]. The genus *Salmonella* comprises two species, *Salmonella bongori* and *Salmonella enterica*. *Salmonella enterica* is further divided into six subspecies, among which *Salmonella enterica* subsp. enterica is the most clinically important and most frequently isolated [5, 6]. Rapid identification of the causative pathogen is therefore essential for effective surveillance, diagnosis, treatment, prevention, and control of foodborne disease outbreaks [7].

The conventional method for *Salmonella* subtyping relies on agglutination reactions between specific antisera and the isolate’s somatic (O), flagellar (H), and capsular Vi antigens. Although accurate, this approach is labor-intensive, time-consuming, and requires the use of numerous antisera [8]. Novel technologies, such as matrix-assisted laser desorption ionization time-of-flight mass spectrometry (MALDI-TOF MS), are widely used for bacterial identification. This library-based method enables rapid, reliable, and cost-effective species-level identification with high sensitivity and specificity [9, 10] by analyzing mass-to-charge (m/z) profiles of microbial proteins and peptides [11]. However, serotype-level identification remains challenging and requires further development [9]. These limitations are largely attributable to overlapping ribosomal protein mass profiles, limited spectral diversity, and underrepresentation of rare *Salmonella* serotypes in existing MALDI-TOF MS reference databases. Although MALDI-TOF MS has been used for more than 20 years, the literature evaluating its accuracy for identifying *Salmonella* serotypes remains limited [12]. Establishing serotype-level accuracy would increase confidence in the technology and support its complementary use alongside conventional subtyping methods in epidemiological and clinical settings, enabling faster reporting.

Antimicrobial agents are commonly used to treat salmonellosis; however, inappropriate or excessive use may contribute to the development of antimicrobial resistance (AMR). AMR in *Salmonella* is an escalating threat to public health, food security, and the global economy. If effective interventions are not implemented, AMR could cause millions of deaths annually, with projections reaching up to 10 million deaths by 2050 [13, 14]. Therefore, natural compounds such as *trans*-cinnamic acid (TCA) have been explored because of their antimicrobial potential. TCA is a phenylpropanoid found in cinnamon bark and various plants and exhibits a broad range of biological activities, including antibacterial, antifungal, anti-inflammatory, antioxidant, and anticancer effects [15–17]. TCA exerts antimicrobial activity by damaging bacterial cell membrane integrity in both Gram-positive and Gram-negative bacteria, leading to cell lysis, and by inhibiting key biosynthetic pathways, including protein and enzyme synthesis, required for bacterial viability [18]. In addition, TCA has been reported to reduce the viability of *Salmonella* Typhimurium in contaminated food, indicating its potential as a natural preservative and an alternative to conventional antimicrobial agents [19].

Although MALDI-TOF MS is widely adopted for rapid species-level identification of *Salmonella* spp., its routine application for reliable serotype discrimination remains limited. Most existing studies focus on prevalent serotypes and rely primarily on library-based identification, which is constrained by overlapping ribosomal protein spectra and incomplete reference databases, particularly for region-specific and less common serotypes. Consequently, there is insufficient validation of serotype-level accuracy using objective performance metrics and reproducible biomarker peaks. In parallel, while AMR in *Salmonella* spp. continues to intensify, the integration of rapid diagnostic tools with non-antibiotic antimicrobial strategies has been inadequately explored. Natural compounds such as TCA have demonstrated antimicrobial activity against *Salmonella* spp.; however, systematic evaluation of their inhibitory and bactericidal effects against multiple poultry-associated serotypes, alongside advanced diagnostic approaches, remains scarce. This gap limits the development of coordinated surveillance and control strategies that simultaneously address rapid detection and AMR mitigation within the poultry chain.

The present study aimed to address these gaps by evaluating the accuracy of MALDI-TOF MS for serotype discrimination of *Salmonella* spp. isolated from the poultry chain through specific peak analysis and comparison with conventional subtyping. In addition, the antimicrobial effectiveness of TCA was assessed by determining minimum inhibitory concentration (MIC) and minimum bactericidal concentration (MBC) values against representative *Salmonella* serotypes, including *Salmonella* Enteritidis, *S*. Typhimurium, and *Salmonella* Kentucky, as well as other less common serotypes. By integrating serotype-specific spectral characterization with antimicrobial susceptibility profiling, this study sought to provide evidence supporting a dual approach that links rapid serotype identification with non-antibiotic intervention strategies for improved *Salmonella* surveillance, control, and AMR risk reduction in poultry production systems.

## MATERIALS AND METHODS

### Ethical approval

This study was conducted in accordance with the Animal Care guidelines of Kasetsart University and was approved by the Institutional Biosafety Committee (Approval No. IBC-66-V12). Permission for sample collection was obtained from all participating poultry farms before study initiation. All experimental procedures involving *Salmonella* spp. were performed under Biosafety Level 2 containment in compliance with applicable biosafety and laboratory safety regulations.

### Study period and location

The study was conducted from May 2023 to February 2024 at the Kamphaeng Saen Veterinary Diagnostic Center, Faculty of Veterinary Medicine, Kasetsart University, Kamphaeng Saen, Nakhon Pathom, Thailand, and the Department of Veterinary Technology, Faculty of Veterinary Technology, Kasetsart University, Bangkok, Thailand. Samples were collected from broiler production systems in the central and eastern regions of Thailand.

### Study design and sample size

This study was designed as a cross-sectional observational study. A total of 63 samples from two broiler farms during the study period were selected by convenience sampling based on sample availability. The study had two main objectives. Objective 1 was to evaluate the performance of MALDI-TOF MS for the identification and differentiation of *Salmonella* serotypes compared with the conventional subtyping method. Objective 2 was to determine the MIC and MBC of TCA against the *Salmonella* isolates obtained in this study.

### Sample collection and isolation of *Salmonella*

A total of 63 samples were obtained from the Kamphaeng Saen Veterinary Diagnostic Center and collected from two chicken farms, with 30 samples from Farm 1 and 33 samples from Farm 2. Samples included surface swabs, rectal swabs, boot swabs, rice hulls, box liners, paper padders, ceca, mouse feces, chicken breast without skin salted (SBB), and boneless leg (BL).

Surface swabs were collected from environmental surfaces in poultry houses (floors, walls, and equipment) using two sterile cotton swabs, covering approximately 100 cm², and the swabs were added to 5 mL of buffered peptone water (BPW). Rectal swabs were collected using sterile cotton swabs, and five swabs were added into 10 mL of BPW. Boot swabs were collected from disposable boot covers worn while walking through poultry houses to sample litter-associated contamination. Solid samples, including rice hulls, box liners, paper padders, and meat samples, were collected aseptically, and 25 g of each sample was added to 225 mL of BPW. All samples were transported to the laboratory at 4°C.

For pre-enrichment, samples were mixed with 5–225 mL of BPW, depending on the sample type, using a stomacher at maximum speed for 2 min and then incubated at 37°C for 16–20 h. After pre-enrichment, 100 µL of each culture was inoculated onto modified semisolid Rappaport–Vassiliadis agar and incubated at 41.5°C for 18–24 h. Growth from modified semisolid Rappaport–Vassiliadis agar was streaked onto xylose lysine deoxycholate agar and brilliant green agar, followed by incubation at 37°C for 18–24 h. Presumptive isolates were confirmed using biochemical tests, including triple sugar iron agar, urease test, and L-lysine decarboxylation test.

### Bacterial isolates and reference strains

A total of 63 field *Salmonella* isolates obtained from poultry-related samples were analyzed. The isolates comprised six serotypes: *S*. Enteritidis, *S*. Typhimurium, *S*. Kentucky, *Salmonella* Newjersey, *Salmonella* Fresno, and *Salmonella* Weltevreden (Table 1). These field isolates were used for MALDI-TOF MS-based identification and serotype-specific peak analysis, as well as for TCA antimicrobial susceptibility testing.

**Table 1:** *Salmonella* spp. strains isolated from the poultry chain.

Source	Serotype (number of isolates)
Surface swab	*Salmonella* Kentucky (1)
Rectal swab	*S.* Kentucky (2)
Boot swab	*Salmonella* Enteritidis (21), *Salmonella* Typhimurium (6), *S.* Kentucky (7), *Salmonella* Fresno (1), *Salmonella* Weltevreden (1)
Rice hull	*S.* Typhimurium (1), *S.* Kentucky (1)
Box liner	*S.* Enteritidis (3), *S.* Typhimurium (4)
Paper padder	*S.* Enteritidis (1), *S.* Typhimurium (1)
Ceca	*S.* Enteritidis (3)
Mouse feces	*Salmonella* Newjersey (1)
Chicken breast without salted skin	*S.* Enteritidis (8)
Boneless leg	*S.* Enteritidis (1)

In addition to field isolates, reference strains were included to support method validation and comparative analyses. The reference strains used in this study were *S*. Typhimurium American Type Culture Collection (ATCC) 13311 and ATCC 14028 (ATCC, Manassas, VA, USA), *S*. Enteritidis DMST 15676, and *S*. Kentucky DMST 62216 (Department of Medical Sciences, Nonthaburi, Thailand). These reference strains were used as controls for MALDI-TOF MS spectral profiling and for comparison of MIC and MBC determinations.

### Preservation of isolates

Pure cultures of confirmed *Salmonella* isolates were preserved for long-term storage. In brief, isolates were grown overnight in tryptic soy broth (TSB) at 37°C to reach the late exponential to early stationary phase. For cryopreservation, 500 µL of the TSB culture was mixed with TSB containing 30% glycerol, and the mixture was stored at −20°C until further analysis. The storage duration ranged up to approximately 4 years prior to MALDI-TOF MS analysis and antimicrobial susceptibility testing.

### Identification of *Salmonella* by MALDI-TOF MS

*Salmonella* spp. isolates preserved in 30% glycerol were streaked onto tryptic soy agar (TSA), and reference strains (*S*. Typhimurium ATCC 13311, *S*. Typhimurium ATCC 14028, *S*. Enteritidis DMST 15676, and *S*. Kentucky DMST 62216) were streaked onto blood agar. All cultures were incubated at 37°C for 18–24 h. A single colony from each culture was selected and smeared onto a VITEK® MS 48-well target slide (bioMérieux, Marcy-l’Étoile, France). The direct smear method was used.

To ensure analytical reproducibility, each isolate was spotted in duplicate. The mass spectra were acquired in linear positive ion mode using the VITEK® MS system. The laser intensity was adjusted to obtain processed spectra with a minimum acceptable signal intensity of 30 mV. Each spectrum consisted of accumulated profiles, with one profile corresponding to five laser shots. A target of 100 acceptable profiles per spot was applied, with a minimum of 30 good profiles. Subsequently, 1 µL of α-cyano-4-hydroxycinnamic acid matrix solution was overlaid and allowed to completely dry at room temperature. The matrix solution consisted of 28% acetonitrile, with 3.10 g of α-cyano-4-hydroxycinnamic acid dissolved in 100 mL of solvent. Once dried, the target slide was inserted into the instrument.

Instrument calibration was performed prior to sample analysis using a fresh culture of *Escherichia coli* ATCC 8739 as the calibration standard. Calibration was based on comparison of measured time-of-flight values with a predefined reference mass list of ribosomal protein masses and was accepted only when the measured masses matched the reference values within the manufacturer-defined tolerance limits. Species-level identification was accepted only when the system generated a single, consistent identification with high confidence across replicate spots. Isolates yielding multiple identifications, low-confidence results, or inconsistent outcomes between replicates were considered unreliable and excluded from further analysis.

### MALDI-TOF MS spectral acquisition and data analysis

The mass spectral profile was analyzed using Spectral Archive and Microbial Identification System (SARAMIS) software version 4.1.0 with database KB 4.17, operated in research use only mode. Identification confidence levels were interpreted according to SARAMIS classification criteria. A “Good” identification was defined as a single taxonomic choice with a probability ranging from 60% to 99.9%. Results classified as “Low discrimination” were defined by two to four possible choices with a cumulative probability of 100%, and further confirmatory testing was required. Isolates yielding no significant choice or more than four possible choices with a cumulative probability of 100% were classified as “No identification” or “Inconclusive identification” and were excluded from downstream analysis.

Mass spectral profile analysis was confined to the 2,000–20,000 Da range because this range predominantly detects ribosomal proteins, which are highly stable and minimally affected by culture conditions [20]. These characteristics make them reliable biomarkers for accurate microbial identification. The mass spectra of the reference strains were compared with those of the isolated samples. Automated peak picking was performed using default SARAMIS parameters, including baseline subtraction, normalization, and selection of reproducible peaks based on signal-to-noise ratio and relative peak intensity. Peak data were analyzed as relative abundance rather than absolute intensity.

### Specific peak analysis using the SARAMIS database

Specific peak analysis was performed using the SARAMIS database to identify serotype-associated mass peaks through comparative evaluation of mass spectral profiles between field isolates and their corresponding reference strains. For each serotype, the presence of peaks was assessed across multiple field isolates and technical replicates to evaluate reproducibility and consistency.

A serotype-specific mass peak was defined when it was consistently detected in the majority of isolates (≥70%) belonging to the same serotype, reproducible across replicate spectra, and absent or inconsistently detected in isolates of other serotypes. Peaks that fulfilled these criteria were retained for further analysis.

To ensure analytical robustness, peak comparisons were restricted to spectra that met predefined quality acceptance criteria. Outlier values were identified during peak comparison when detected m/z values fell outside the expected serotype-associated peak range or showed inconsistent detection across technical replicates. Such outliers were excluded from peak calculations before comparative analysis.

### Preparation of TCA

TCA (CAS No. 140-10-3; 97% purity) was purchased from Fluka (Buchs, Switzerland). A total of 0.148 g of TCA was weighed and dissolved in 1,000 µL of dimethyl sulfoxide (DMSO; CAS No. 67-68-5; 99.9% purity), with complete solubility ensured by vortex mixing at room temperature, to prepare a stock solution. Subsequently, the stock solution was subjected to two-fold serial dilutions to obtain concentrations of 20, 10, 5, 2.5, 1.25, 0.625, 0.312, and 0.156 mM. The prepared solutions were stored at 4°C, protected from light, and used within 2 weeks of preparation. The final DMSO concentration in each well was 0.0156% (v/v). All other chemicals used in this study were of analytical grade.

### Determination of MIC

Gram-negative *S*. Enteritidis, *S*. Typhimurium, *S*. Kentucky, *S*. Newjersey, *S*. Fresno, and *S*. Weltevreden were used in this study. All strains were prepared by inoculating a single colony into 30 mL of TSB and incubated at 37°C for 22 h under shaking at 250 rpm. The absorbance of each bacterial strain was measured at 595 nm using a spectrophotometer, adjusted to 0.1–0.2 with TSB, and diluted 1:100 to achieve a final concentration of 0.001–0.002.

The MIC of TCA was determined using the broth dilution method with modifications in accordance with the Clinical and Laboratory Standards Institute M07™ guideline. Different concentrations of TCA were used. Two microliters of TCA dissolved in DMSO were added to 98 µL of each bacterial suspension into polystyrene microtiter plates to achieve concentrations of 20, 10, 5, 2.5, 1.25, 0.625, 0.312, and 0.156 mM, respectively. The lowest concentration of TCA required to visibly inhibit growth was considered the MIC.

A mixture of 2 µL of DMSO and 98 µL of bacterial suspension served as the positive control, while a mixture of 2 µL of DMSO and 98 µL of TSB served as the negative control. The microtiter plates were incubated at 37°C for 22 h. The absorbance of each plate was measured at 595 nm using a microplate reader. Then, 50 µL of *p*-iodonitrotetrazolium violet (INT) was added to indicate bacterial growth at all concentrations of TCA. Samples were incubated at 37°C for 30 min. All MIC assays were performed using three technical replicates and were repeated across ten independent biological replicates.

### Determination of MBC

The overnight culture of bacteria from MIC determination was used. A volume of 5 µL was removed from each well, streaked onto TSA plates, and incubated at 37°C for 22 h. The MBC was defined as the lowest concentration of TCA at which bacterial growth was not observed. All MBC determinations were conducted using three technical replicates and ten biological replicates, consistent with the MIC assay design.

### Controls used in MIC and MBC assays

Quality control in MIC and MBC assays was ensured by including appropriate growth and sterility controls. The positive control consisted of 2 µL of DMSO mixed with 98 µL of bacterial suspension in TSB, adjusted to an initial inoculum of approximately optical density 0.001–0.002. This control consistently showed visible turbidity after incubation, confirming normal bacterial growth in the absence of TCA.

The negative control consisted of 2 µL of DMSO mixed with 98 µL of sterile TSB without bacterial inoculation, which remained clear throughout incubation, confirming medium sterility and absence of contamination.

A solvent control containing a bacterial suspension with DMSO at the same final concentration used in test wells but without TCA was included to evaluate potential solvent effects. No inhibitory effect of DMSO on bacterial growth was observed, indicating that any growth inhibition detected was attributable to TCA rather than the solvent. Visible turbidity was interpreted as bacterial growth, whereas a clear appearance indicated growth inhibition.

### Statistical analysis

The performance of MALDI-TOF MS was evaluated using overall percentage agreement (OPA), positive percentage agreement (PPA), negative percentage agreement (NPA), and Cohen’s kappa statistic, with corresponding 95% confidence intervals. MIC and MBC values were summarized as mean ± standard deviation. Differences in MIC and MBC values among *Salmonella* serotypes were evaluated using the non-parametric Kruskal–Wallis test due to non-normal data distribution. All statistical analyses were two-tailed, and p < 0.05 was considered statistically significant. Statistical analyses were performed using Statistical Package for the Social Sciences software version 26.0 (IBM Corp., Armonk, NY, USA). MIC and MBC data processing and descriptive statistical analyses were performed using Microsoft Excel 365.

## RESULTS

### Identification and distribution of *Salmonella* strains

*Salmonella* strains were isolated from various sources along the poultry production chain, including surface swab (n = 1), rectal swab (n = 2), boot swab (n = 36), rice hull (n = 2), box liner (n = 7), paper padder (n = 2), ceca (n = 3), mouse feces collected from the poultry house (n = 1), SBB (n = 8), and BL (n = 1) (Table 1).

The highest detection frequency of *Salmonella* was observed in boot swab samples, whereas the lowest detections were recorded in surface swab, mouse feces, and BL samples. Boot swab samples accounted for the majority of isolates (36/63, 57.1%), while surface swab, mouse feces, and BL samples each represented 1.6% of the total isolates.

### Mass spectral profile analysis and identification of specific biomarker peaks

#### Comparative MALDI-TOF MS profiling of *Salmonella* serotypes

**Figure 1:**
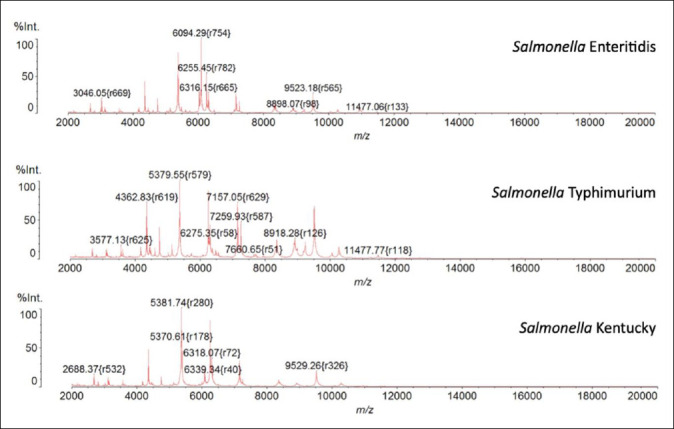
Mass spectral profiles of *Salmonella* Enteritidis, *Salmonella* Typhimurium, and *Salmonella* Kentucky serotypes showing the mass-to-charge ratio (m/z).

MALDI-TOF MS analysis of isolates and reference strains, including *S*. Enteritidis DMST 15676, *S*. Typhimurium ATCC 13311, *S*. Typhimurium ATCC 14028, and *S*. Kentucky DMST 62216, revealed highly similar mass spectral profiles. These results indicate conserved protein expression patterns among the major *Salmonella* serotypes, particularly associated with ribosomal proteins (Figure 1).

Mass spectral profiles of *Salmonella* Enteritidis, *Salmonella* Typhimurium, and *Salmonella* Kentucky serotypes showing the mass-to-charge ratio (m/z).

Specific biomarker peaks were identified by comparing the mass spectra of each isolate with those of the corresponding reference strains. Previous studies by Jirapanth *et al*. [21] reported characteristic peaks at 6094 ± 2 m/z for *S*. Enteritidis, 7156 ± 2 m/z for *S*. Typhimurium, and 5370 ± 2 m/z for *S*. Kentucky, which were consistently detected in all isolates of their respective serotypes (Table 2). This comparative approach confirmed that these specific peaks were unique to each serotype and absent in others.

**Table 2:** Specific mass spectral peaks of *Salmonella* serotypes isolated from the poultry production chain as determined by MALDI-TOF MS.

*Salmonella* serotype^a^	n	Mean (m/z)	Mode (m/z)	Range (m/z)
*Salmonella* Enteritidis^b^	37	6094.82	6095.87	6092.37–6096.63
*Salmonella* Typhimurium	12	7157.12	7157.96	7154.71–7158.59
*Salmonella* Kentucky	11	5370.55	5370.50	5368.78–5372.07

MALDI-TOF MS = Matrix-assisted laser desorption ionization time-of-flight mass spectrometry,

a No specific peaks were detected for *Salmonella* Newjersey, *Salmonella* Fresno, and *Salmonella* Weltevreden,

b The outlier value of 7157.82 m/z, which did not correspond to the specific peak and was possibly due to analytical error, was excluded from the calculation.

The SARAMIS software recorded only the presence of biomarker peaks and did not provide intensity or relative abundance data.

Overlay spectral analysis for serotype discrimination

**Figure 2:**
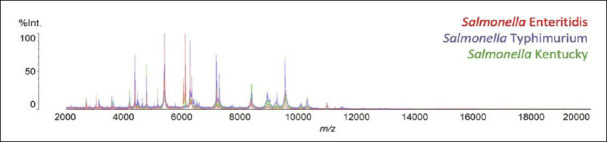
Overlay of mass spectral profiles of *Salmonella* Enteritidis, *Salmonella* Typhimurium, and *Salmonella* Kentucky obtained using Matrix-assisted laser desorption ionization time-of-flight mass spectrometry.

To further visualize inter-serotype spectral similarity and discriminatory features, the mass spectral profiles of the three major serotypes were overlaid (Figure 2).

Overlay of mass spectral profiles of *Salmonella* Enteritidis, *Salmonella* Typhimurium, and *Salmonella* Kentucky obtained using Matrix-assisted laser desorption ionization time-of-flight mass spectrometry.

The overlaid spectra demonstrated a high degree of similarity across serotypes, reflecting conserved ribosomal protein profiles within the genus *Salmonella*. Despite this substantial overlap, distinct serotype-associated biomarker peaks were consistently observed across multiple isolates and technical replicates and were absent in non-corresponding serotypes. These reproducible differences support the application of peak-based discrimination for serotype-level identification using MALDI-TOF MS.

#### Agreement between MALDI-TOF MS and conventional subtyping

Overall, MALDI-TOF MS exhibited high accuracy in identifying *Salmonella* serotypes when compared with conventional subtyping methods (Table 3). Specificity was evaluated using NPA, while OPA and PPA were also calculated. Agreement between methods was further assessed using Cohen’s kappa coefficient.

**Table 3:** Comparison of results obtained using MALDI-TOF MS and the conventional subtyping method for *Salmonella* serotypes.

*Salmonella* Serotype^a^	Number of Isolates^b^	MALDI-TOF MS^c^				OPA^d^	PPA^d^	NPA^d^	Cohen’s kappa

		SE	ST	SK	another serotype				
*Salmonella* Enteritidis	37	36	1	0	0	98.4%	97.3%	100%	0.65
*Salmonella* Typhimurium	12	0	12	0	0	100%	100%	100%	0.93
*Salmonella* Kentucky	11	0	0	11	0	100%	100%	100%	0.93
Other serotype	3	0	0	0	3	100%	100%	100%	1.00

a SE = *Salmonella* Enteritidis, ST = *Salmonella* Typhimurium, SK = *Salmonella* Kentucky; other serotypes include *Salmonella Newjersey, Salmonella* *Fresno*, and *Salmonella* Weltevreden. Each serotype was analyzed using n isolates, with four technical replicates per isolate (total spectra = n × 4).

b Number of isolates identified using conventional subtyping

c Number of isolates identified using MALDI-TOF MS

d The OPA, PPA, and NPA values are presented with corresponding 95% confidence intervals.

MALDI-TOF MS = Matrix-assisted laser desorption ionization time-of-flight mass spectrometry.

Most *S*. Enteritidis isolates were correctly identified, although one isolate was misclassified as *S*. Typhimurium. In contrast, all isolates of *S*. Typhimurium, *S*. Kentucky, and the group of other serotypes, including *S*. Newjersey, *S*. Fresno, and *S*. Weltevreden, were correctly identified.

The inclusion of rare serotypes provided novel spectral information not commonly available in commercial MALDI-TOF MS databases. These serotypes lacked distinct biomarker peaks, likely due to underrepresentation in reference libraries, highlighting existing gaps in serotype-specific biomarker coverage.

Cohen’s kappa values indicated substantial agreement for *S*. Enteritidis (0.65), near-perfect agreement for *S*. Typhimurium and *S*. Kentucky (0.93), and perfect agreement for the other serotypes (1.00). OPA, PPA, and NPA ranged from 97.3% to 100%, confirming the reliability of MALDI-TOF MS for *Salmonella* serotype identification.

### MIC of TCA

MIC represents the lowest concentration of an antimicrobial agent that completely inhibits visible bacterial growth. It was determined by measuring absorbance using a microplate reader, with growth confirmation performed using INT.

The MIC values for *S*. Enteritidis, *S*. Typhimurium, *S*. Kentucky, *S*. Newjersey, *S*. Fresno, and *S*. Weltevreden demonstrated complete growth inhibition at a concentration of 10 mM, as indicated by the absence of color change.

### MBC of TCA

MBC represents the lowest concentration of an antimicrobial agent that completely eliminates bacterial viability. It was determined by culturing samples from 96-well plates containing TCA concentrations of 20, 10, 5, 2.5, 1.25, 0.625, 0.312, and 0.156 mM onto TSA plates, including appropriate controls.

The MBC values for all tested *Salmonella* serotypes showed complete bacterial killing at 20 mM, with no growth observed on agar plates.

All serotypes exhibited identical MIC and MBC values for TCA, with no statistically significant differences among serotypes (p > 0.05) (Table 4).

**Table 4:** Minimum inhibitory concentration and minimum bactericidal concentration values for *trans*-cinnamic acid on *Salmonella* Enteritidis, *Salmonella* Typhimurium, *Salmonella* Kentucky, *Salmonella* Newjersey, *Salmonella* Fresno, and *Salmonella* Weltevreden.

Serotype	Minimum inhibitory concentration (mM)	Minimum bactericidal concentration (mM)
*Salmonella* Enteritidis	10.0 ± 0.0^a^	20.0 ± 0.0^a^
*Salmonella* Typhimurium	10.0 ± 0.0^a^	20.0 ± 0.0^a^
*Salmonella* Kentucky	10.0 ± 0.0^a^	20.0 ± 0.0^a^
*Salmonella* Newjersey	10.0 ± 0.0^a^	20.0 ± 0.0^a^
*Salmonella* Fresno	10.0 ± 0.0^a^	20.0 ± 0.0^a^
*Salmonella* Weltevreden	10.0 ± 0.0^a^	20.0 ± 0.0^a^

a Statistically significant difference (*p* > 0.05). Means of triplicates ± standard deviation

Kruskal–Wallis analysis further confirmed that the distribution of MIC and MBC values did not differ significantly among serotype categories (p > 0.05), indicating comparable antimicrobial susceptibility to TCA across the tested *Salmonella* serotypes.

## DISCUSSION

### Distribution of *Salmonella* serotypes in poultry production systems

The *Salmonella* serotypes identified in this study are consistent with previous reports from poultry production systems in Southeast Asia, where *S*. Enteritidis and *S*. Typhimurium are commonly detected, and *S*. Kentucky is frequently reported as a dominant serotype. The detection of less common serotypes, such as *S*. Newjersey, *S*. Fresno, and *S*. Weltevreden, further reflects regional diversity and highlights the importance of surveillance approaches capable of detecting both prevalent and emerging serotypes within the poultry chain.

### Performance of MALDI-TOF MS for serotype identification

This study demonstrates the strong potential of MALDI-TOF MS for identifying *Salmonella* serotypes in the poultry production chain [12, 20, 22]. The high levels of agreement between MALDI-TOF MS and conventional subtyping methods, with OPA, PPA, and NPA values ranging from 97.3% to 100%, indicate that MALDI-TOF MS can be effectively integrated into routine diagnostic procedures for epidemiological surveillance and outbreak investigations [12, 23]. However, the slightly lower agreement observed for *S*. Enteritidis (κ = 0.65) compared with *S*. Typhimurium and *S*. Kentucky (κ = 0.93) suggests that certain serotypes may share similar mass spectral patterns, which can occasionally lead to misidentification [5, 21].

Misclassification observed in one *S*. Enteritidis isolate may be attributed to biological and technical factors inherent to MALDI-TOF MS-based serotyping. Variations in protein expression, particularly ribosomal proteins that dominate MALDI-TOF MS spectra, may alter peak intensities or result in missing diagnostic peaks at the strain level. In addition, *S*. Enteritidis shares highly conserved protein profiles with closely related serotypes, which can produce overlapping spectral features and reduce discriminatory power. Furthermore, limited strain diversity in reference spectral libraries may contribute to misclassification when field isolates exhibit atypical or underrepresented protein patterns. These factors emphasize the need for expanded reference databases and complementary subtyping approaches when interpreting MALDI-TOF MS serotype assignments. Similar overlaps related to shared ribosomal proteins or biomarker peaks have been reported previously [11, 24]. Expanding spectral databases with additional reference strains, particularly epidemiologically important serotypes, would help reduce this variability [12, 25].

### Serotype-specific peak discrimination and database limitations

The detection of specific peaks at 6094 ± 2 m/z for *S*. Enteritidis, 7156 ± 2 m/z for *S*. Typhimurium, and 5370 ± 2 m/z for *S*. Kentucky demonstrates that peak-based discrimination is feasible. These findings are consistent with those reported by Jirapanth *et al*. [21] and Yang *et al*. [24], who showed that incorporating serotype-specific peaks into MALDI-TOF MS workflows can improve subtyping accuracy. In contrast, the absence of identifiable peaks for *S*. Newjersey, *S*. Fresno, and *S*. Weltevreden highlights serotype-specific biomarker gaps for less common serovars, providing insight into serotype-level spectral variation and the current limitations of MALDI-TOF MS peak detection algorithms.

These gaps are most likely attributable to limited representation of uncommon serotypes in commercial MALDI-TOF MS reference databases, which are often biased toward prevalent serotypes from Europe and North America. This bias may reduce discriminatory accuracy for field isolates originating from Southeast Asia. Expansion of reference spectral libraries and integration of machine learning-based approaches that analyze full-spectrum patterns may improve discrimination among closely related serotypes and support the development of region-specific diagnostic tools [12, 19, 26].

### Antimicrobial activity of TCA against *Salmonella*

This study also evaluated the antimicrobial activity of TCA against all *Salmonella* serotypes. The observed MIC (10 mM) and MBC (20 mM) values indicate consistent inhibitory and bactericidal effects across all isolates. Although these MIC and MBC values are relatively high compared with conventional antimicrobials, higher localized concentrations may be feasible in certain applications, supporting the potential role of TCA as a complementary control measure in poultry production and food safety systems. Such applications may include surface treatments or processing steps where exposure is spatially and temporally limited, allowing higher effective concentrations without systemic application.

Future studies may explore formulation strategies, including encapsulation, stabilization, or nano-formulations, to enhance the antimicrobial efficacy of TCA at lower effective concentrations. These findings align with previous reports demonstrating that cinnamic acid derivatives exert antimicrobial effects by disrupting cell membrane integrity, altering permeability, and inhibiting essential biosynthetic pathways [15, 17, 18, 27]. Although effective concentrations were relatively high compared with synthetic antimicrobials, the natural origin and broad-spectrum activity of TCA make it an attractive candidate for use as a food preservative or alternative antimicrobial agent, particularly for AMR mitigation [13, 28, 29]. This study demonstrates the consistent inhibitory effect of TCA across all tested serotypes, supporting its potential application as a natural preservative to reduce reliance on conventional antibiotics in poultry production.

### Integrated control strategy and study limitations

The integration of MALDI-TOF MS for rapid serotyping with TCA as a natural antimicrobial offers a combined approach for controlling *Salmonella* in poultry production systems. Rapid and accurate serotype identification enables timely epidemiological interventions, while plant-based antimicrobials may reduce dependence on conventional antibiotics and limit the emergence of resistant strains. Within a hurdle-based control framework, TCA may also be used synergistically with other non-antibiotic interventions, such as organic acids, sanitation measures, or physical treatments, thereby enhancing overall efficacy while minimizing reliance on high concentrations of a single agent.

Several limitations should be acknowledged. No standard AQC strain was included in this study. In addition, MALDI-TOF MS performance depends on database completeness; therefore, rare or emerging serotypes may not be accurately detected without regular updates [12, 22]. Moreover, the in vitro nature of antimicrobial assays may not fully represent in vivo conditions, where food matrix composition, pH, and storage conditions may influence antimicrobial effectiveness [19, 30]. Future research should focus on field validation of MALDI-TOF MS subtyping protocols, expansion of spectral libraries, optimization of TCA formulations to achieve effective doses, and evaluation of synergistic effects with other natural antimicrobials or physical treatments [18, 31, 32]. We propose a dual-control strategy in which rapid MALDI-TOF MS-based serotyping is paired with MIC/MBC screening of plant-based antimicrobials, establishing an integrated and rapid response framework for poultry chain surveillance and foodborne pathogen control.

## CONCLUSION

This study demonstrated that MALDI-TOF MS, when combined with specific peak analysis, provides high accuracy for *Salmonella* serotype identification within the poultry chain. Strong agreement with conventional subtyping was observed, with OPA, PPA, and NPA values ranging from 97.3% to 100%, and substantial to near-perfect concordance for major serotypes. Serotype-associated peaks were consistently identified for *S*. Enteritidis, *S*. Typhimurium, and *S*. Kentucky, confirming the feasibility of peak-based discrimination. In parallel, TCA showed consistent antimicrobial activity against all tested serotypes, with uniform MIC and MBC values, indicating broad-spectrum inhibitory and bactericidal effects.

The findings support the integration of MALDI-TOF MS into routine poultry diagnostics for rapid serotype-level surveillance and outbreak investigation. Rapid identification enables timely epidemiological responses, while the demonstrated antimicrobial activity of TCA highlights its potential as a complementary, non-antibiotic control measure. Such applications may be particularly relevant for localized interventions in food processing or surface treatments, contributing to food safety enhancement and AMR mitigation.

Key strengths include the combined evaluation of rapid diagnostic technology and a natural antimicrobial agent, the inclusion of multiple poultry-associated *Salmonella* serotypes, and the use of objective performance metrics to assess serotype identification accuracy. The identification of serotype-specific mass spectral peaks further strengthens the evidence for extending MALDI-TOF MS beyond species-level identification.

Several limitations should be acknowledged. MALDI-TOF MS performance remains dependent on database completeness, which may restrict discrimination of rare or region-specific serotypes. In addition, antimicrobial activity was assessed in vitro, and the observed MIC and MBC values may not fully reflect effectiveness under in vivo or food matrix conditions. The absence of a standard AQC strain is also recognized as a limitation.

Future studies should focus on expanding MALDI-TOF MS reference spectral libraries with regionally relevant serotypes, refining peak-based algorithms, and incorporating advanced analytical approaches such as full-spectrum or machine-learning-based classification. Further research is also warranted to optimize TCA formulations to enhance efficacy at lower concentrations and to evaluate synergistic effects with other non-antibiotic interventions under practical production and processing conditions.

Overall, this study supports a dual-control strategy that combines rapid MALDI-TOF MS-based serotyping with MIC/MBC screening of plant-derived antimicrobials. This integrated approach provides a practical framework for strengthening *Salmonella* surveillance, improving food safety, and reducing reliance on conventional antibiotics in poultry production systems.

## DATA AVAILABILITY

All the generated data are included in the manuscript.

## AUTHORS’ CONTRIBUTIONS

NJ, PU, and AS: conceptualization, resources, funding acquisition, and writing of the original draft. PU, KV, and AS: review and editing. NJ, JT, SR, NT, and AS: methodology. NJ and AS: data curation, formal analysis, statistical analysis, and visualization. PU and AS: supervision. All authors have read and approved the final version of the manuscript.

## COMPETING INTERESTS

The authors declare that they have no competing interests.

## PUBLISHER’S NOTE

Veterinary World remains neutral with regard to jurisdictional claims in the published institutional affiliations.

## References

[1] Yang S. M, Baek J, Kim E, Kim H. B, Ko S, Kim D, Yoon H, Kim H.Y (2020) Development of a genoserotyping method for *Salmonella* Infantis detection on the basis of pangenome analysis. Microorganisms.

[2] Kasturi K. N, Drgon T (2017). Real-time PCR method for detection of *Salmonella* spp. in environmental samples. Appl. Environ. Microbiol.

[3] Ma B, Li J, Chen K, Yu X, Sun C, Zhang M ((2020)). Multiplex recombinase polymerase amplification assay for the simultaneous detection of three foodborne pathogens in seafood. Foods.

[4] World Health Organization Regional Office for South-East Asia (2016) Burden of foodborne diseases in the South-East Asia region. World Health Organization, Regional Office for South-East Asia, New Delhi, India.

[5] Dieckmann R, Malorny B ((2011)). Rapid screening of epidemiologically important *Salmonella* enterica subsp. enterica serovars by whole-cell matrix-assisted laser desorption ionization time-of-flight mass spectrometry. Appl. Environ. Microbiol.

[6] Park S. H, Kim H. J, Cho W. H, Kim J. H, Oh M. H, Kim S. H, Lee B. K, Ricke S. C, Kim H. Y ((2009)). Identification of *Salmonella* enterica subspecies I and serovars Typhimurium, Enteritidis, and Typhi using multiplex PCR. FEMS Microbiol. Lett.

[7] Sabat A. J, Budimir A, Nashev D, Sá-Leão R, van Dijl J, Laurent F, Grundmann H, Friedrich A.W ((2013)). Overview of molecular typing methods for outbreak detection and epidemiological surveillance. Euro Surveill.

[8] Grimont P. A. D, Weill F. X ((2007)). Antigenic formulae of the *Salmonella* serovars.

[9] Kliem M, Sauer S ((2012)). The essence on mass spectrometry-based microbial diagnostics. Curr. Opin. Microbiol.

[10] Christner M, Trusch M, Rohde H, Kwiatkowski M, Schlüter H, Wolters M, Aepfelbacher M, Hentschke M ((2014)). Rapid MALDI-TOF mass spectrometry strain typing during a large outbreak of Shigatoxigenic Escherichia coli. PLoS One.

[11] Sandrin T. R, Goldstein J. E, Schumaker S ((2013)). MALDI-TOF MS profiling of bacteria at the strain level:A review. Mass Spectrom. Rev.

[12] Persad A. K, Fahmy H. A, Anderson N, Cui J, Topalcengiz Z, Jeamsripong S, Spanninger P. M, Buchanan R. L, Kniel K. E, Jay-Russell M. T, Danyluk M. D, Rajashekara G, LeJeune J.T ((2022)). Identification and subtyping of *Salmonella* isolates using matrix-assisted laser desorption ionization time-of-flight mass spectrometry. Microorganisms.

[13] Antimicrobial Resistance Collaborators (2022) Global burden of bacterial antimicrobial resistance in 2019:A systematic analysis Lancet.

[14] Ha D. R, Haste N. M, Gluckstein D. P ((2019)). The role of antibiotic stewardship in promoting appropriate antibiotic use. Am. J. Lifestyle Med.

[15] Sova M ((2012)). Antioxidant and antimicrobial activities of cinnamic acid derivatives. Mini Rev. Med. Chem.

[16] Huang Y, Zeng F, Xu L, Zhou J, Liu X, Le H ((2013)). Anticancer effects of cinnamic acid in lung adenocarcinoma cell line H1299-derived stem-like cells. Oncol. Res.

[17] Jitareanu A, Tataringa G, Zalaru A. M, Stanescu U ((2011)). Toxicity of some cinnamic acid derivatives to common bean (Phaseolus vulgaris). Not. Bot. Horti Agrobot. Cluj Napoca.

[18] Guzman J. D ((2014)). Natural cinnamic acids, synthetic derivatives and hybrids with antimicrobial activity. Molecules.

[19] Pina-Pérez M, Martínez A, Rodrigo D ((2012)). Cinnamon antimicrobial effect against *Salmonella* Typhimurium cells treated by pulsed electric fields in pasteurized skim milk beverage. Food Res. Int.

[20] Dingle T. C, Butler-Wu S. M ((2013)). MALDI-TOF mass spectrometry for microorganism identification. Clin. Lab. Med.

[21] Jirapanth N, Tuangrithaiwanich J, Rakmit S, Thongdee N, Vongkamjan K, Kovitvadhi A, Sanguankiat A ((2024)). subtyping *Salmonella* Enteritidis, Kentucky, and Typhimurium by specific peak analysis using MALDI-TOF MS. Thai J. Vet. Med.

[22] Ren J, Xia J, Zhang M, Liu C, Xu Y, Wu J, Li Y, Zhou M, Li S, Cao W ((2025)). Automated identification of *Salmonella* serotype using MALDI-TOF mass spectrometry and machine learning techniques. J. Clin. Microbiol.

[23] Gao A, Fischer-Jenssen J, Slavic D, Rutherford K, Lippert S, Wilson E, Chen S, Leon-Velarde C. G, Martos P ((2023)). Rapid identification of *Salmonella* serovars Enteritidis and Typhimurium using MALDI-TOF MS coupled with multivariate analysis and artificial intelligence. J. Microbiol. Methods.

[24] Yang S. M, Kim E, Kim D, Baek J, Yoon H, Kim H.Y ((2021)). Rapid detection of *Salmonella* Enteritidis, Typhimurium, and Thompson by specific peak analysis using MALDI-TOF MS. Foods.

[25] Costa A, Catalano F, Alcain A, Panagopulo M, Riquel Moyelak J. E, Brengi S, Moroni M, Viñas M R ((2025)). Rapid discrimination of *Salmonella* Enteritidis from other serovars with MALDI-TOF MS in Argentina. Rev. Argent. Microbiol.

[26] Akimowicz M, Bucka-Kolendo J ((2020)). MALDI-TOF MS application in food microbiology. Acta Biochim. Pol.

[27] Kabat M, Popiół J, Gunia-Krzyżak A ((2024)). Cinnamic acid derivatives as potential multifunctional agents in cosmetic formulations. Molecules.

[28] Letsididi K, Lou Z, Letsididi R, Mohammed K, Maguy B ((2018)). Antimicrobial and antibiofilm effects of *trans*-cinnamic acid nanoemulsion and its potential application on lettuce. LWT Food Sci. Technol.

[29] Yilmaz S, Sova M, Ergün S ((2018)). Antimicrobial activity of *trans-*cinnamic acid and commonly used antibiotics against fish pathogens. J. Appl. Microbiol.

[30] Singhal N, Kumar M, Kanaujia P. K, Virdi J. S ((2015)). MALDI-TOF mass spectrometry:An emerging technology for microbial identification. Front. Microbiol.

[31] Kang L, Li N, Li P, Zhou Y, Gao S, Gao H, Xin W, Wang J ((2017)). MALDI-TOF MS accuracy in *Salmonella* identification at species level. Eur. J. Mass Spectrom. (Chichester).

[32] Annuur R. M, Triana D, Ernawati T, Murai Y, Aswad M, Hashimoto M, Tachrim Z.P ((2024)). Review of cinnamic acid skeleton modification for antibacterial derivatives. Molecules.

